# A Hybrid LPG/CFBG for Highly Sensitive Refractive Index Measurements

**DOI:** 10.3390/s120607318

**Published:** 2012-05-30

**Authors:** An Sun, Zhishen Wu

**Affiliations:** International Institute for Urban Systems Engineering, Southeast University, Nanjing 210096, Jiangsu, China; E-Mail: zswu@ibaraki.ac.jp

**Keywords:** optical fiber sensor, refractive index, hybrid FBG/LPG, cladding modes

## Abstract

A simple and high sensitive method employing a hybrid long period grating (LPG)/chirped fiber Bragg grating (CFBG) for refractive index (RI) measurements is proposed and investigated experimentally. The wide wavelength range of backward cladding modes are excited through the coupling and recoupling between LPG and CFBG. Experimental results indicate that the recoupled cladding modes between LPG and CFBG and core mode are modulated by the surrounding RI and highly sensitive RI measurements can be achieved by simply measuring the reflected intensity changes of the recoupled cladding modes and core mode.

## 1. Introduction

Cladding mode-based fiber optical refractometers have been widely studied as a promising solution for long term environment monitoring or chemical applications due to their high sensitivity to surrounding RI changes. So far various kinds of optical fiber grating-based RI sensors that employ LPG [1–3], FBG [4–11] or Tilted FBG [12–17] (TFBG) have been proposed. For most reported fiber grating RI sensors, their shortcomings lie in that usually an expensive and accurate spectrum analysis system is needed. Moreover, in many schemes, special fiber structures such as thinned or etched fibers [4,5], D-fibers [6] or microstructure fibers [7–9] are employed to enhance cladding mode coupling and recoupling efficiency and thus to improve the sensitivity of the refractometer. Recently, some schemes that employ TFBG [18–23] have been reported for RI measurement in which a special fiber is employed for the recoupling of the cladding mode from cladding to fiber core. The RI can then be detected by measuring the reflected intensity of the recoupled cladding mode. Although it provides a comparatively simple method, the recoupling power of cladding modes is so weak that its sensitivity is seriously affected by the poor recoupling efficiency of cladding modes. Moreover, hybrid grating structures [20–23] have also been investigated for RI measurement, in which the cladding modes coupled by LPG can be recoupled into the fiber core through FBG. Its disadvantages, however, also lie in a limited sensitivity since only a small part of the cladding modes can be recoupled due to the narrow bandwidth of FBG. Additionally, the requirements for a highly accurate spectrum analyzer in these schemes also limit the applications of hybrid gratings for RI measurement.

CFBG have been reported previously for sensing applications [24–26]. Like FBG, however, it is core mode based grating sensors that are intrinsically immune to outer RI changes. Therefore, so far no research has been conducted concerning the application of CFBG for RI measurements. In this paper, a simple but highly sensitive scheme employing a hybrid LPG/CFBG for cost-effective RI measurement is proposed for the first time. Strong reflected cladding modes with a wide wavelength range can be excited and recoupled into the fiber core with a high efficiency through the hybrid LPG/CFBG. By simply detecting the power of the enhanced cladding modes, highly sensitive RI measurements can be achieved through the proposed sensor structure.

## 2. Sensing Principle

**Figure 1.**
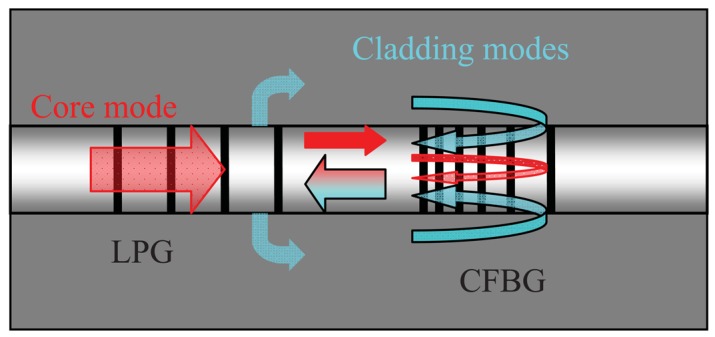
Schematic diagram of hybrid LPG/CFBG refractometer.

The basic principle is the wide wavelength range cladding mode coupling and recoupling between LPG and CFBG as shown in Figure 1. Both LPG and CFBG are written on hydrogenated telecom SMF 28 fiber using a 248 nm excimer laser. Two gratings are spliced together and the transmission fiber length between two gratings is about 1 cm.

When light transmits to the LPG section, the wavelength that meets the LPG resonance condition is coupled into the cladding layer of fiber, forming the forward cladding mode. The cladding mode can propagate to the CFBG section with little loss because of the short transmission distance between LPG and CFBG. In the CFBG section, part of the cladding modes are recoupled into the fiber core. Because CFBG have a wide reflection spectrum range, all the recoupled cladding modes that meet the CFBG resonance condition are reflected as counter-propagation cladding modes. The coupling and recoupling mechanism of cladding modes between LPG and FBG has been introduced and analyzed in [20,22]. For our proposed hybrid LPG/CFBG, it can be treated as an array of hybrid LPG/FBG pairs in which all of these hybrid grating pairs have a uniform LPG resonance, but sequentially different FBG resonances. The reflected spectrum of each hybrid grating pair consists of a FBG resonance peak and a series of recoupled cladding modes that correspond to the highly effective index cladding mode as a resonance of FBG period. As a result, the reflected LPG/CFBG spectrum is the overlap of a series of core modes and recoupled cladding modes that correspond to different LPG/FBG pairs. By this way, the recoupling coefficient of cladding modes from cladding layer to fiber core can be greatly improved. When the surrounding RI changes, the cladding modes are attenuated through the evanescence field between cladding and the outer medium. Because of the high cladding mode recoupling efficiency between LPG and CFBG, any RI changes can be sensitively detected by measuring the RI induced intensity change of the reflected cladding modes.

**Figure 2.**
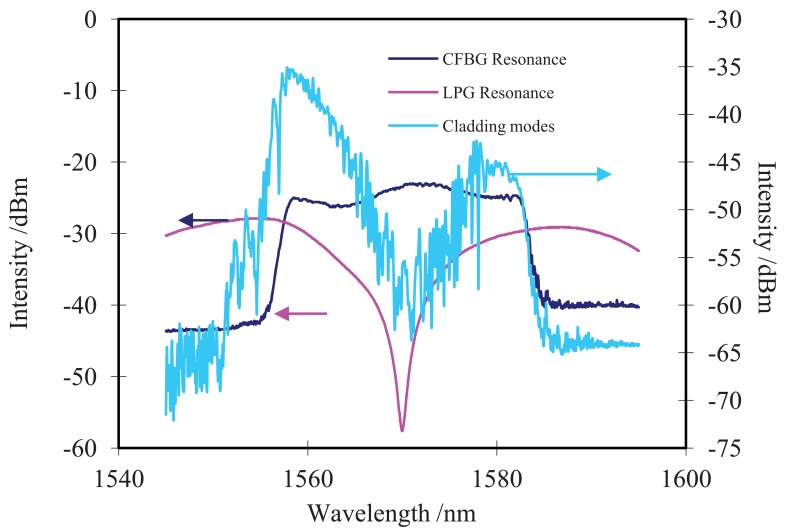
Spectra of LPG and CFBG before and after splicing.

The corresponding spectrum of annealed LPG and CFBG before and after splicing is observed through an MS 9740C optical spectrum analyzer (OSA) as illustrated in Figure 2. The grating length of both LPG and CFBG are around 2 cm. The central wavelength and 3 dB bandwidth of LPG is about 1,565 nm and 16 nm respectively. The period of LPG is around 450 μm.

The CFBG has the fundamental Bragg resonance at 1,570 nm and a bandwidth of 20 nm at 3 dB. Both the dip transmission loss of LPG and the average reflectivity of CFBG are around 90%. Light emitted from a broadband amplified spontaneous emission (ASE) source is launched into the hybrid LPG/CFBG through a three-port fiber circulator. The reflected light is measured through OSA with a wavelength resolution of 0.03 nm. As shown in Figure 2, strong cladding modes with a wide wavelength range from 1,550 nm to 1,585 nm are excited. Compared with the initial spectrum of CFBG, its short wavelength side range is broadened to 1,550 nm, increasing about 5 nm due to the highly effective index cladding mode as a resonance of the chirped grating period. It can also be seen in Figure 2 that the reflected intensity of the hybrid LPG/CFBG (right side vertical axes) is greatly weakened compared with that of CFBG (left side vertical axes) before splicing with the LPG. As we described above, the CFBG can be treated as a series of FBG with different resonance conditions, which means that the low order cladding modes of shorter wavelength FBG are overlapped with the high order cladding modes that correspond to the longer wavelength FBG resonance, so the whole reflected spectrum of cladding modes is continuous with irregular fluctuation and the order of cladding modes can hardly be distinguished due to the superimposition of cladding modes that correspond to different FBG resonances. Additionally, the reflected spectrum of the hybrid grating also includes a weak core mode due to the reflection of CFGB, which is overlapped with the cladding modes.

## 3. Experimental Results and Discussion

**Figure 3.**
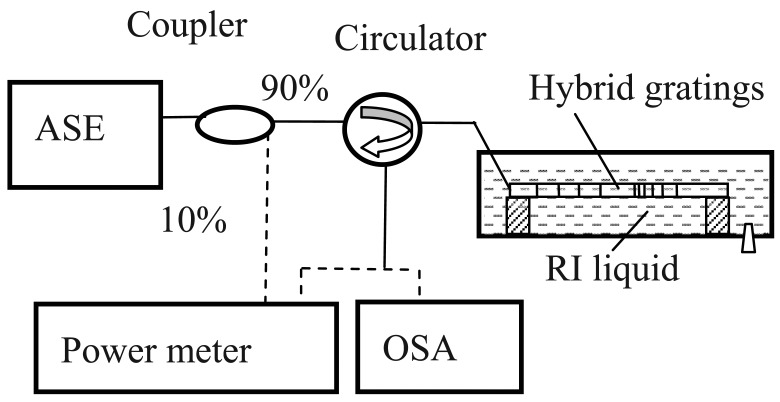
Experimental setup of hybrid LPG/CFBG for RI measurement.

Experiment is carried on to test the performance of the hybrid LPG/CFBG for RI measurement as shown in Figure 3. The whole fiber sensor structure is kept straight with small pre-strain to avoid any bending-induced spectrum changes. The pre-strain is less than 100 με, which is so small that the corresponding spectrum change of the hybrid gratings can be neglected.

**Figure 4.**
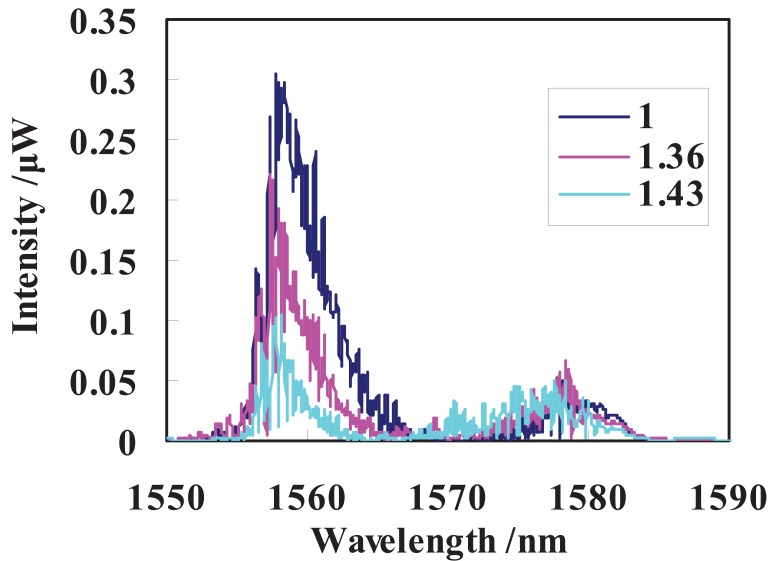
Reflected spectrum changes of hybrid LPG/CFBG at different RI.

Ten percent of light emitted from the ASE source is used as reference light through a 1:9 coupler to compensate the light source fluctuation and another 90% is used as sensing light. Both a power-meter with a 1 nW resolution and the OSA are employed to measure the corresponding power and spectra changes of the cladding modes. The sensor is immersed from air (RI = 1.00) into liquids with different RI alues ranging from 1.33 to 1.43 in sequence and the corresponding response of the hybrid LPG/CFBG is measured and shown in Figure 4. During the experiment, the room temperature is kept at 24 °C to avoid any temperature change induced spectrum change of hybrid grating.

Following with the increasing of RI, the intensity of reflected cladding modes degrades monotonically attenuating through the evanescent field between cladding and surrounding medium according to Figure 4. In fact, the increasing RI affects the reflected spectrum of the hybrid LPG/CFBG in two aspects. Firstly, the LPG resonance shifts toward the shorter wavelength side. Secondly, cladding modes are attenuated through the evanescence field between cladding and RI liquid. If we take the unevenness of the CFBG spectrum into consideration, the reflected intensity of the hybrid grating should increase following with the blue shift of the LPG because the CBFG has a higher reflectivity on the longer wavelength side, as shown in Figure 2. If the unevenness of the CFBG is neglected, the reflected intensity should remain unchanged. When the RI changes from 1.00–1.43, the LPG resonance is still within the spectrum range of the CFBG, which shifts around 5 nm according to our previous experimental results, so theoretically the whole reflected power modulated by the shift of LPG resonance should change little or increase slightly. Moreover, if the shift of LPG is responsible for the observed effect, the intensity of reflected spectrum at longer wavelength side should increase greatly following with the increasing of RI due to the blue shift of LPG resonance. But it can be seen in Figure 4 that the intensity increasing of reflected spectrum at longer wavelength side is so small that can be neglected. Therefore, according to the experimental results, we can get the conclusion that the modulation effect of outer RI to recoupled cladding modes through the evanescence field between cladding and outer medium is dominate. Following with the increasing of RI, the cladding modes within the full bandwidth of reflected spectrum are attenuated and only very weak core mode is immune from RI.

**Figure 5.**
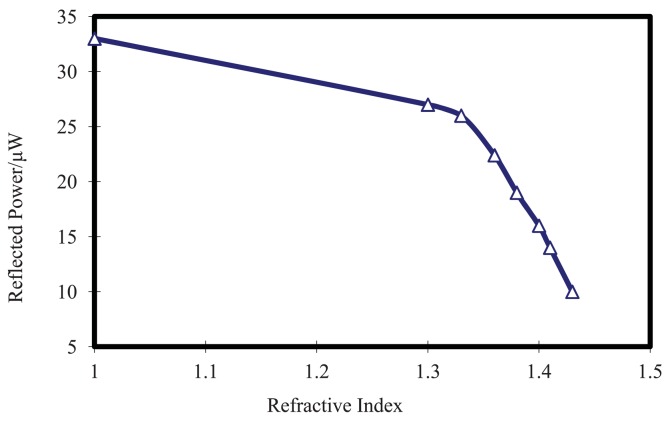
Reflected power of hybrid LPG/CFBG *versus* RI.

The corresponding response of reflected cladding modes power to RI as shown in Figure 5 can be divided into two sections. When RI increases gradually from 1.00 to 1.36, the reflected power of cladding modes decreases from 33 μW to 22.4 μW, that corresponding to a sensitivity of 29 μW/RIU (Refractive Index Unit). With the RI increase further, the intensity of cladding modes is attenuated drastically, which corresponding to a power decreasing from 22.4 μW to 10 μW when RI increase gradually from 1.36 to 1.43. In this response section, a higher sensitivity about 177 μW/RIU can be achieved. This nonlinear sensitivity is primarily caused by the different sensitivities of lower order cladding modes and high order cladding modes to RI. For hybrid grating structure, low order cladding modes are dominate, while the intensity of high order cladding mode are comparatively weak. High order cladding modes are more sensitive to low RI change. So when RI is lower than 1.40, only high order cladding modes are modulated and the corresponding RI sensitivity is comparatively low. When RI exceed 1.40, more and more low order cladding modes are affected by RI through the evanescence field between cladding and outer medium. Therefore, the sensitivity of hybrid LPG/CFBG within this RI range is greatly enhanced.

Considering that 1 nW power resolution can be easily achieved through power-meter, the principal highest RI resolution for proposed hybrid LPG/CFBG is about 5.6 × 10^−6^ RIU within the RI range from 1.36 to 1.43. Even at the RI of 1.00–1.36, the sensitivity can reach 3.4 × 10^−5^ RIU. In [18], a TFBG-based RI sensor, in which the reflected cladding modes of TFBG can be recoupled from cladding to fiber core through a misaligned fiber structure has been proposed. The sensitivity they have obtained, which is only 1.1 μW/RIU, was serious affected due to the limited cladding mode recoupling efficiency. By employing the proposed hybrid LPG/CFBG structure, the recoupling efficiency of cladding modes within a wide wavelength range can be greatly enhanced as we have shown. Experimental results also confirm that the corresponding sensitivity of our scheme is about thirty times higher than that previously reported in [18].

In many practical applications, usually the RI is around 1.33, that is, close to the RI of water. For our proposed hybrid LPG/FBG sensors, the sensitivity is comparatively low. This indicates that an optimal design is needed to enhance the recoupling coefficiency of high order cladding modes for the sensitivity improvement of the hybrid LPG/CFBG refractometer at the low RI range. Theoretically, it can be achieved through optimal chirped designs such as tilted or thinned LPG/CFBG structures and further experiment about these is ongoing.

## 4. Conclusions

In conclusion, a simple and high sensitive refractometer based on a hybrid LPG/CFBG is described and investigated. Due to the high cladding mode coupling and recoupling efficiency between LPG and CFBG, strong cladding modes with a wide wavelength range can be effectively excited and observed by employing the proposed hybrid LPG/CFBG structure. By measuring the RI-induced reflected intensity changes of the cladding modes, highly sensitive RI measurements can be achieved while the need for complex and costly spectrum interrogation systems is effectively avoided.

## References

[1] Patrick H.J., Kersey A.D., Bucholtz F. (1998). Analysis of the response of long period fiber gratings to external index of refraction. J. Lightwave Technol..

[2] Villar I.D., Matias I.R., Arregui F.J. (2005). Enhancement of sensitivity in long-period fiber gratings with deposition of low-refractive index materials. Opt. Lett..

[3] Rindorf L., Bang O. (2008). Highly sensitive refractometer with a photonic-crystal-fiber long-period grating. Opt. Lett..

[4] Iadicicco A., Cusano A., Cutolo A., Bernini R., Giordano M. (2004). Thinned fiber Bragg gratings as high sensitivity refractive index sensor. IEEE Photon. Technol. Lett..

[5] Chen N., Yun B., Cui Y. (2006). Cladding mode resonances of etch-eroded fiber Bragg grating for ambient refractive index sensing. Appl. Phys. Lett..

[6] Zhou K., Chen X., Zhang L., Bennion I. (2004). High sensitivity optical chemsensor based on etched D-fibre Bragg gratings. Electron. Lett..

[7] Iadicicco A., Campopiano S., Cutolo A., Giordano M., Cusano A. (2005). Refractive index sensor based on microstructured fiber Bragg grating. IEEE Photon. Technol. Lett..

[8] Huy M.C.P., Laffont G., Frignac Y., Dewynter-Marty V., Ferdinand P., Roy P., Blondy J.M., Pagnoux D., Blanc W., Dussardier B. (2006). Fibre Bragg grating photowriting in microstructured optical fibres for refractive index measurement. Meas. Sci. Technol..

[9] Huy M.C.P., Laffont G., Dewynter V., Ferdinand P., Roy P., Auguste J., Pagnoux D., Blanc W., Dussardier B. (2007). Three-hole microstructured optical fiber for efficient fiber Bragg grating refractometer. Opt. Lett..

[10] Wu Q., Semenova Y., Yan B., Ma Y., Wang P., Yu C., Farrell G. (2011). Fiber refractometer based on a fiber Bragg grating and single-mode–multimode–single-mode fiber structure. Opt. Lett..

[11] Liang W., Huang Y.Y., Xu Y., Lee R.K., Yariv A. (2005). Highly sensitive fiber Bragg grating refractive index sensors. App. Phys. Lett..

[12] Allsop T., Neal R., Rehman S., Webb D.J., Mapps D., Bennion I. (2007). Generation of infrared surface plasmon resonances with high refractive index sensitivity utilizing tilted fiber Bragg gratings. Appl. Opt..

[13] Shevchenko Y.Y., Albert J. (2007). Plasmon resonances in gold-coated tilted fiber Bragg gratings. Opt. Lett..

[14] Chryssis A.N., Lee S.M., Lee S.B., Saini S.S., Dagenais M. (2005). High sensitivity evanescent field fiber Bragg grating sensor. IEEE Photon. Technol. Lett..

[15] Chen X.F., Zhou K.M., Zhang L., Bennion I. (2005). Optical chemsensor based on etched tilted Bragg grating structures in multimode fiber. IEEE Photon. Technol. Lett..

[16] Huy M.C.P., Laffont G., Dewynter V., Ferdinand P., Labonté L., Pagnoux D., Roy P., Blanc W., Dussardier B. (2006). Tilted fiber Bragg grating photowritten in microstructured optical fiber for improved refractive index measurement. Opt. Express.

[17] Laffont G., Ferdinand P. (2001). Tilted short-period fibre-Bragg grating induced coupling to cladding modes for accurate refractometry. Meas. Sci. Technol..

[18] Guo T., Tam H.Y., Krug P.A., Alert J. (2009). Reflective tilted fiber Bragg grating refractometer based on strong cladding to core recoupling. Opt. Express.

[19] Guo T., Chen C., Laronche A., Albert J. (2008). Power-referenced and temperature-calibrated optical fiber refractometer. IEEE Photon. Technol. Lett..

[20] Zhang A.P., Tao X., Chung W.H., Guan B.O., Tam H.Y. (2002). Cladding-mode-assisted recouplings in concatenated long-period and fiber Bragg gratings. Opt. Lett..

[21] Caucheteur C., Debliquy M., Lahem D., Mégret P. (2008). Hybrid fiber gratings coated with a catalytic sensitive layer for hydrogen sensing in air. Opt. Express.

[22] Han M., Guo F.W., Lu Y.F. (2010). Optical fiber refractometer based on cladding-mode Bragg grating. Opt. Lett..

[23] Shao L.Y., Laronche A., Smietana M., Mikulic P., Bock W.J., Albert J. (2010). Highly sensitive bend sensor with hybrid long-period and tilted fiber Bragg grating. Opt. Commun..

[24] Okabe Y., Tsuji R., Takeda N. (2004). Application of chirped fiber Bragg grating sensors for identification of crack locations in composites. Composites Part A.

[25] Yashiro S., Okabe T., Toyama N., Takeda N. (2007). Monitoring damage in holed CFRP laminates using embedded chirped FBG sensors. Int. J. Solids Struct..

[26] Swart P.L., Lacquet B.M., Chtcherbakov A.A. (2005). Chirped fiber Bragg grating sensor for pressure and position sensing. Opt. Eng..

